# Contemporary approaches and treatment perspectives for chronic scrotal content pain: insights from a national practice patterns survey of reproductive urologists

**DOI:** 10.1038/s41443-025-01101-x

**Published:** 2025-06-04

**Authors:** Anthony Bettencourt, Bryan D. Naelitz, Leila Momtazi-Mar, Amelia Khoei, Joseph Borrell, Jaxon R. Jeffery, Thiago P. Furtado, Catherine S. Nam, Nahid Punjani, Scott D. Lundy, Sriram V. Eleswarapu

**Affiliations:** 1 David Geffen School of Medicine, University of California, Los Angeles, CA 90095 USA; 2 Glickman Urological and Kidney Institute, Cleveland Clinic, Cleveland, OH 44195 USA; 3 Cleveland Clinic Lerner College of Medicine, Case Western Reserve University, Cleveland, OH 44195 USA; 4 Department of Urology, Michigan Medicine, University of Michigan, Ann Arbor, MI 48109 USA; 5 Mayo Clinic Alix School of Medicine, Phoenix, AZ 85050 USA; 6 Division of Andrology, Department of Urology, David Geffen School of Medicine at UCLA, Los Angeles, CA 90095 USA; 7 Department of Urology, Mayo Clinic, Phoenix, AZ 85054 USA

**Keywords:** Surgery, Diagnosis

## Abstract

A 25-item electronic survey was circulated to reproductive urologists from around the US to evaluate current national diagnostic and therapeutic practices for the management of chronic scrotal content pain (CSCP). Questions addressed physician demographics, referral patterns, diagnostic protocols, treatment approaches, and outcome perceptions. Forty-one of 183 (22.4%) invited participants completed the survey. Among ten conservative treatment options, reassurance (41.5%) and NSAIDs (31.7%) were most frequently ranked first, while opioid medications were the least preferred, with 56.1% of respondents ranking them last of the ten options. Microsurgical denervation of the spermatic cord was the most commonly utilized surgical procedure overall (95.1%) and the first choice for post-vasectomy pain syndrome (PVPS) in 63.0% of respondents. Vasectomy reversal was the surgery of choice for PVPS in only 22.0% of respondents. Respondents perceived higher complete symptom resolution (median: 75% vs. 25%) and lower failure rates (median: 10% vs. 20%) in patients who had surgery versus those who had only conservative measures. Only 7.3% of participants reported using validated assessment tools such as the Chronic Orchialgia Symptom Index. These results demonstrate that wide variability persists in CSCP management among reproductive urologists, though there is consensus that surgery has a higher success rate as compared to conservative management.

## Introduction

Chronic scrotal content pain (CSCP) is defined as three or more months of scrotal content pain that interferes with daily life [1, 2]. Available data suggests CSCP accounts for nearly 1% of primary care and up to 5% of outpatient urology visits, and is estimated to affect nearly 100,000 men in the US each year [3–5]. As of April 2025, the American Urological Association (AUA) released its first clinical guidelines for the diagnosis and management of CSCP [6–8]. This comes as welcome relief, as the absence of formal recommendations has led to diverse and inconsistent management strategies, with some patients seeing an average of four to five urologists in their pursuit of symptom relief [9].

The etiology of CSCP is often multifactorial and/or idiopathic, with many patients undergoing extensive clinical testing. Conventional clinical evaluation requires a thorough history and physical examination, and may include laboratory testing and imaging such as scrotal ultrasound. Structural etiologies of CSCP include pathologies of the epididymis, tunica vaginalis, testicle, and spermatic cord. Referred pathologies, such as chronic pelvic pain syndrome, pelvic floor dysfunction, obstructing ureteral calculi, and hip and spinal disorders can also cause scrotal discomfort. Up to 40–50% of cases of CSCP are found to be idiopathic [2, 10]. Treatment ranges from conservative approaches, including pharmacologic therapy and supportive underwear, to surgical interventions such as microsurgical denervation of the spermatic cord (MDSC), vasectomy reversal (if pain related to a prior vasectomy), epididymectomy, or orchiectomy.

While outcomes for individual treatments have been studied, few investigations have explored how urologists, particularly fellowship-trained reproductive urologists, approach CSCP in practice. This remains a difficult patient population to treat, and given the prior lack of standardized clinical guidelines, we hypothesized that there is substantial variation in practice patterns in the evaluation and treatment of CSCP. Accordingly, we developed and administered a survey to assess CSCP practice patterns among fellowship-trained reproductive urologists to help promote awareness and provider dialogue surrounding CSCP for the benefit of patients who struggle with this condition, and to reduce unnecessary burden to our patients and healthcare system.

## Methods

A survey characterizing the diagnosis and management of CSCP was designed by the authors from multiple institutions. All senior authors are fellowship-trained reproductive urologists and part of a multi-institutional infertility research consortium. After iterative revisions, the senior authors reached consensus on a 25-item survey (Supplementary Figure 1).

The survey was administered from January 24, 2025, to February 24, 2025. It was distributed to reproductive urologists from around the US. The questionnaire was administered through Qualtrics (Provo, Utah), with all responses anonymized. Each respondent could only participate once, based on a unique link. Survey queries assessed respondents’ experience managing patients presenting with CSCP, along with demographic data regarding the level of training, board certification, and years in practice. Information was collected regarding referral sources, diagnostic testing strategies, treatment modalities (both surgical and conservative), and patient outcomes. Finally, respondents were asked open-ended questions regarding challenges they face in managing patients with CSCP and recommended areas for further research. Only those respondents who fully completed the survey were included in the analysis.

Descriptive statistics, utilizing means and medians with interquartile ranges (IQR), were calculated. Comparisons were performed using the Mann-Whitney U-test for continuous numeric variables and the chi-square test or Fisher’s exact test for categorical variables. The query regarding the number of years in practice was stratified into two groups for comparative analysis: ≤5 years and >5 years. A significance threshold of p < 0.05 was used for all statistical tests. All statistical analyses were performed using R Statistical Software (R v4.4.3) [11].

## Results

### Physician demographics & patient volume

Of the 183 physicians surveyed, 57 responded (31.1%), and 41 completed the survey in full (22.4%) and were included in the final analysis. All respondents were practicing urologists with subspecialty training, and the majority were board-certified (82.9%). There were nearly equal numbers of respondents in practice ≤5 years (48.8%) and >5 years (51.2%). Full demographic and practice characteristics are presented in Table 1.

**Table 1:** Respondent demographics and practice characteristics.

Respondent characteristic	N (%)
Total physicians surveyed	183
Survey respondents	57 (31.1%)
Surveys completed and included in analysis	41 (22.4%)
**Specialty**	
Practicing urologist with subspecialty training	41 (100%)
**Board certification status**	
Board-certified	34 (82.9%)
In process of certification	7 (17.1%)
**Annual CSCP Patient Volume**	
Less than 10	0.0%
10–49	13 (32.5%)
50–100	13 (32.5%)
Over 100	14 (35.0%)
**Years in practice**	
<1 year	3 (7.3%)
1–5 years	17 (41.5%)
6–10 years	9 (22%)
11–20 years	7 (17.1%)
>20 years	5 (12.2%)
**Grouped practice experience**	
*≤5 years*	20 (48.8%)
*>5 years*	21 (51.2%)

### Diagnostic evaluation

Respondents estimated patient referral sources via a sliding scale, with referrals from urologists (median: 30%, IQR: 20–50%), primary care providers (median: 20%, IQR: 10–30%), and self-referrals (median: 15%, IQR: 0–25%) being the three most common sources. They then indicated the three most common diagnostic tests employed by referring providers prior to index evaluation. The top three were scrotal ultrasound (median: 80%, IQR: 50–90%), urinalysis (UA) or urine culture (median: 75%, IQR: 50–100%), and sexually transmitted infection (STI) testing (median: 20%, IQR: 10–50%) (Table 2).

**Table 2:** Diagnostic studies and exam maneuvers completed by referring providers before index evaluation for patients presenting with chronic scrotal content pain.

Study or Exam Maneuver	Completed prior to presentation by referring provider (median %, IQR)
Scrotal ultrasound	80.0%, 50–90%
Urinalysis or urine culture	75.0%, 50–100%
STI testing	20.0%, 10–50%
Hernia exam	10.0%, 0–50%
Advanced Imaging (CT, MRI, etc.)	5.0%, 0–15%
DRE	2.5%, 0–20%
X-ray (hip, low back, etc.)^a^	0.0%, 0–0%
Testicular tumor markers^a^	0.0%, 0–0%
Semen culture^a^	0.0%, 0–0%
Semen analysis^a^	0.0%, 0–0%
Other^a^	0.0%, 0–0%
No testing^a^	0.0%, 0–0%
Inguinal ultrasound^a^	0.0%, 0–0%

*IQR* interquartile range. *STI* sexually transmitted infection, *DRE* digital rectal examination, *CT* computed tomography, *MRI* magnetic resonance imaging.

^a^“0%, 0–0%” reflects a high proportion of “0” responses, meaning that more than 50% of respondents did not report any referrals in that category.

**Fig. 1:**
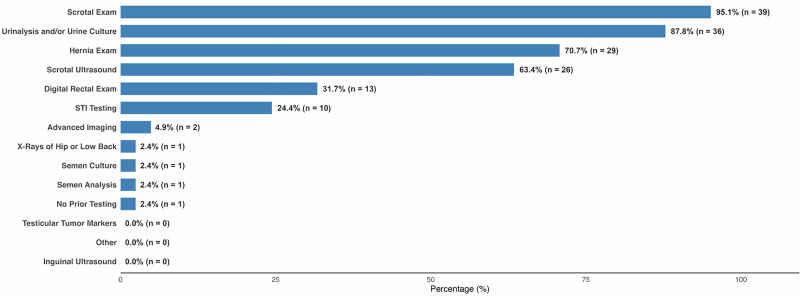
Initial diagnostic tests used over half of the time for index patients presenting with chronic scrotal content pain. *STI* sexually transmitted infection.

When asked which testing strategies they personally performed in over 50% of cases, using a “select all that apply” format, respondents most frequently chose scrotal examination (95.1%, n = 39/41), UA or urine culture (87.8%, n = 36/41), or evaluation for hernia (70.7%, n = 29/41) (Fig. 1). No significant differences were found between physicians with ≤5 years versus >5 years of practice. Overall, respondents noted that the final cause remained idiopathic despite the completion of a comprehensive CSCP workup in a median of 67.0% of cases (IQR: 50–80%).

### Conservative management strategies

**Fig. 2:**
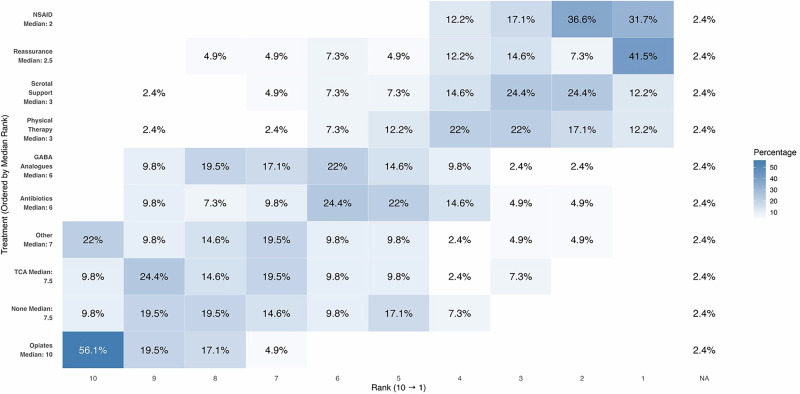
Heatmap distribution of ranked preferences for conservative management of CSCP. The percentage of respondents assigning each rank is displayed in the corresponding cell. Higher intensity color gradation indicates increased preference for that specific rank. The y-axis is ordered from least preferred (10) to most preferred (1). *CSCP* chronic scrotal content pain, *NSAID* non-steroidal anti-inflammatory drug, *TCA* tricyclic antidepressants.

Providers were surveyed on their preference of conservative (non-medical and medical) therapy options including NSAIDs (non-steroidal anti-inflammatory drug), reassurance, scrotal support, pelvic floor physical therapy (PFPT), GABA analogues (gabapentin or pregabalin), antibiotics, tricyclic antidepressants (TCAs), and opioids. Reassurance (41.5%) and NSAIDs (31.7%) were most often ranked as the preferred first-line treatment (Fig. 2). Opioids were consistently ranked among the lowest options with 56.1% of respondents rating them as their least preferred conservative treatment option.

There was a statistically significant difference in the ranking of PFPT with those having ≤5 years in practice assigning a higher ranking compared to those having >5 years in practice (median rank: 3 vs. 4, p = 0.019).

### Spermatic cord nerve block

Providers reported performing spermatic cord nerve blocks in a median of 66.8% (IQR: 40–100%) of patients. In a median of 10.0% (IQR: 0.75–30%) of patients, cord block was used as treatment alone and not for diagnostic purposes.

With regards to the components of the cord block mixture, 85.4% of respondents reported using a short-acting anesthetic, 70.7% reported using a long-acting anesthetic, and 51.2% reported using a steroid. There were no statistically significant differences based on years in practice among inclusion of the various agents (≤5 years vs. >5 years, p = 0.79).

**Fig. 3:**
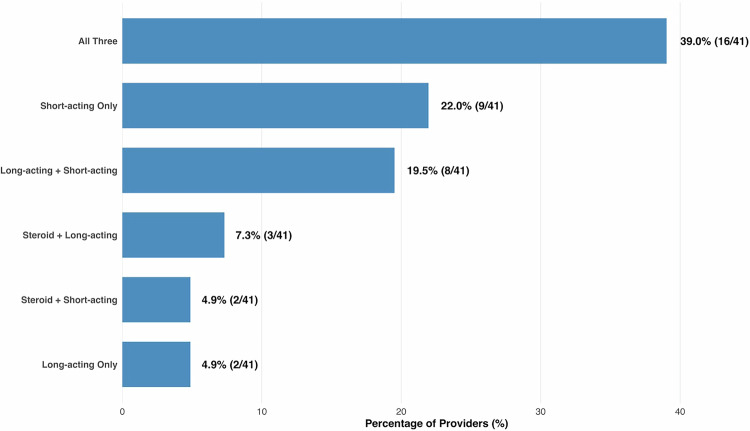
Specific spermatic cord block components and combination usage for CSCP, reported as the percentage of providers who use either a combination or single-agent mixture in their cord block. *CSCP* chronic scrotal content pain.

The most common mixture included agents from all three classes (short-acting anesthetic, long-acting anesthetic, and steroid), reported by 39.0% of respondents (Fig. 3). The next most common mixture was a combination of short- and long-acting anesthetics (22.0%), without steroids. There were no significant differences based on years in practice (≤5 years vs. >5 years, p = 0.35).

### Surgical management strategies

The most common surgical intervention for CSCP was MDSC with 95.1% of respondents reporting they perform this procedure for CSCP. This was followed by varicocelectomy (92.7%), vasectomy reversal (78.0%), epididymectomy (73.2%), orchiectomy (51.2%), and “other” (9.8%). Of the 39 respondents who reported utilizing MDSC for CSCP, providers estimated they performed unilateral MDSC in a median of 80% of cases (IQR: 75–92.5%) and utilized a bilateral approach in 20.0% (IQR: 7.5–25%) of cases. For the 30 respondents who reported performing vasectomy reversals for CSCP, providers estimated that a median of 80.0% (IQR: 50–93.75%) of cases were financed via cash-pay.

### Post-vasectomy pain syndrome management

Respondents were asked which operation they were most likely to perform for the management of post-vasectomy pain syndrome (PVPS). A majority of participants reported MDSC (63.4%) as their preferred surgical option for treating CSCP, followed by vasectomy reversal (22.0%), and epididymectomy (9.8%). One respondent reported using vasectomy revisions for PVPS management and an additional one reported not performing any surgeries for PVPS.

### Treatment outcomes: conservative versus surgical management

On average, participants reported complete resolution of CSCP symptoms in a median of 25.0% (IQR: 10–40%) of their patients following conservative management, and partial resolution occurred in 25.0% (IQR: 20–40%). Treatment failure, or no improvement, was reported in a median of 20.0% (IQR: 15–25%) of patients. 10.0% (IQR: 10–25%) were estimated to be lost to follow-up. In a median of 29.0% (IQR: 15–40%) of patients, conservative management failure led to escalation to surgical intervention.

Surgical management was associated with higher rates of symptom resolution and lower rates of treatment failure or loss to follow-up. On average, respondents reported complete symptom resolution in a median of 70.0% (IQR: 50–80%) of patients, partial resolution in 20.0% (IQR: 10–30%) of patients, and treatment failure in 10.0% (IQR: 5–15%) of patients who underwent surgery. Between 0 and 25% of patients were reported to have been lost to follow-up.

**Fig. 4:**
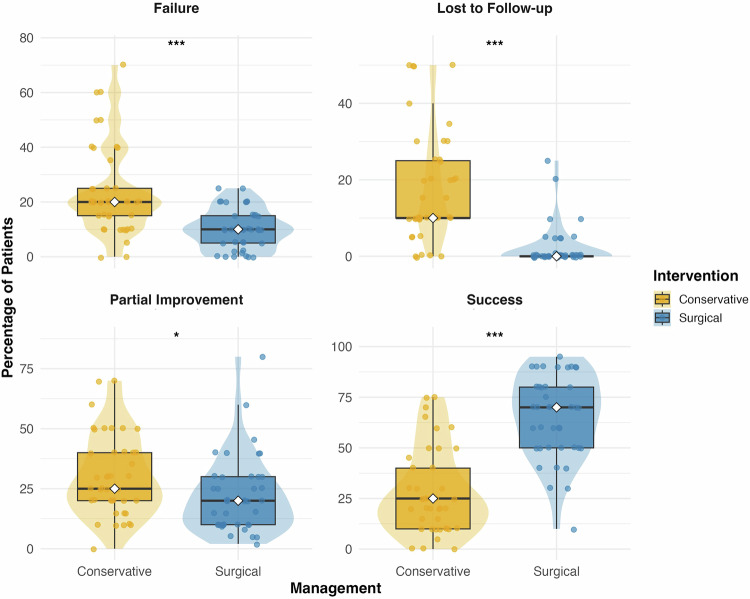
Comparison of provider-reported patient outcomes following conservative (medication and non-medical) versus surgical management for chronic scrotal content pain (CSCP), stratified by outcome category. Each point represents the estimated percentage of patients experiencing a given outcome as reported by an individual provider (n = 41). Violin plots illustrate the distribution of responses, with overlaid boxplots showing the interquartile range and median (white diamond). Statistical comparisons between groups were performed using the Mann–Whitney U test (*p < 0.05, ***p < 0.001), appropriate for non-parametric data. *CSCP* chronic scrotal content pain.

A comparative analysis utilizing the Mann-Whitney U-test was then performed to compare reported outcomes between surgical and conservative management between respondents. Reported rates of complete resolution were significantly higher following surgery compared to conservative management (70% vs. 25%, p < 0.001). Conversely, respondents reported higher rates of only partial resolution (25% vs. 20%, p = 0.011), treatment failure (20% vs. 10%, p < 0.001) and loss to follow-up (10% vs. 0%, p < 0.001) in those who underwent only conservative management (Fig. 4).

### Specialty referrals and measurements of success

To analyze the types and distribution of specialty referrals, respondents were asked to estimate the percentage of referrals made to other, specified specialties. The most common referral reported was to PFPT with a median reported percentage of 75%, (IQR: 40–90%). Second was chronic pain specialty clinic at 18.4% (IQR 5–30%) followed by general surgery at 5.0% (IQR: 0–10%). For referrals to psychiatry, another urologist, or “other” referrals, a large number of respondents reported none, resulting in a median of 0. The mean percentages were 5.9%, 1.4%, and 1.0%, respectively.

Patient-reported subjective outcomes were the most common outcome measure, utilized by 97.6% of respondents in their evaluations. Numeric pain scores were used by 58.5% of respondents, and a reduction in the amount of pain medication used was reported in 41.5%. Very few respondents (7.3%) reported utilizing validated measures for CSCP, such as the Chronic Orchialgia Symptom Index (COSI).

## Discussion

This study provides a comprehensive overview of physician-reported practice patterns for the management of CSCP among reproductive urologists. To our knowledge, this is the first study to directly assess physician preference in the treatment of CSCP. In the context of the first AUA guidelines for CSCP, this survey also provides an important contextualization of baseline practice patterns before their widespread adoption.

Conservative management strategies demonstrated substantial variability, with reassurance and NSAIDs most frequently utilized, whereas opioids were consistently ranked as one of the lowest options by respondents. Other modalities, such as TCAs and GABA analogues, demonstrated wide variability. These results are in line with current AUA guidelines, which recommend acetaminophen, NSAIDs, tricyclic antidepressants, GABA analogues, and non-opioid options as first-line pharmacologic options for CSCP [8]. Despite being recommended by the AUA for CSCP, few studies have evaluated the efficacy of TCAs and GABA analogues. In one study with 26 consecutive patients with CSCP, over 60% of patients on nortriptyline or gabapentin saw a greater than 50% reduction in their pain [12]. Pain improvement was even more pronounced in those with idiopathic CSCP, with 80% of patients achieving a 50% reduction. This same study demonstrated minimal efficacy for these medications in treating PVPS. One randomized controlled trial demonstrated a statistically significant decrease in pain scores with the use of gabapentin for up to three days postoperatively after scrotal surgery [13]. While this study did not directly assess gabapentin for CSCP, it does indicate some efficacy in the reduction of scrotal-related pain. The relatively low preference for TCAs and GABA analogues among respondents in the present study may reflect limited provider familiarity, particularly with TCAs, which are associated with numerous side effects and drug-to-drug interactions [14].

The components of spermatic cord blocks, a mainstay in evaluating primary vs referred CSCP, also varied between providers. A combination of agents, typically a short-acting anesthetic, long-acting anesthetic, and steroid, was most commonly used. To our knowledge, the current research landscape has not yet evaluated the most effective combination of agents for alleviating CSCP. However, given spermatic cord blocks are recommended by the AUA for diagnostic purposes in preparation for surgical management such as MDSC [8], sustained pain relief is not necessarily the primary goal. As seen in our study, most respondents utilized spermatic cord blocks solely for diagnostic purposes. One retrospective cohort study found that spermatic cord blocks with a combination of a long-acting local anesthetic and a steroid provided sustained pain relief in over 70% of men with CSCP [15]. The role of spermatic cord blocks as standalone treatment, with optimal combination of agents, remains an area worthy of further investigation.

MDSC was the most commonly reported surgical intervention for PVPS management. MDSC has been shown to be successful for the treatment of PVPS with success rates as high as 81% when multiple structures (i.e. testes, epididymis, spermatic cord) are involved [16]. Vasectomy reversal for PVPS was reported as the preferred modality by only 22% of respondents, demonstrating guideline non-conformity with AUA guidelines, which specifically recommend vasectomy reversal as the treatment of choice for patients with PVPS. This may be explained in part by the large proportion of respondents who reported self-pay (cash) for their vasectomy reversals, potentially identifying a cost-prohibitive barrier. One study estimated that the median self-pay price for a vasectomy reversal in the U.S. was $2,786, based on publicly available listings of US hospitals [17]. However, this estimate is limited because only 4% of hospitals provided pricing data, and because vasectomy reversal is commonly performed in ambulatory surgery centers or other outpatient sites. A separate study, which included both hospital systems and individual or small group practices, reported a median price of $6500 with a range of $1990 - $14,175 [18]. Future studies should compare the effectiveness of MDSC versus vasectomy reversal for symptomatic improvement, especially considering that in those who wish to preserve sterility, MDSC may be a more suitable option.

Respondents reported that surgical management led to higher complete resolution rates and lower treatment failure rates compared to conservative management. This may reflect a preference toward surgical approaches among fellowship-trained specialists or a perception of complete relief for patients lost to follow-up postoperatively. Prior studies evaluating surgical modalities for the treatment of CSCP have shown varying success rates. MDSC, the most commonly reported surgery used by respondents in our study, has demonstrated success rates of 71–96% when used specifically for treating CSCP [19–22]. In each of these studies, inclusion criteria necessitated failure of conservative management as well as positive response to spermatic cord block. These data, along with the results of our questionnaire, raise the possibility of offering early surgical management in the treatment of CSCP, given that surgery offers a higher likelihood of complete symptom resolution. Future studies should investigate outcomes of early surgical management in patients with CSCP, especially given the low complications associated with MDSC [19–22]. Notably, the newly released AUA guidelines also recommend shared decision-making with the option for early surgical intervention using MDSC or vasectomy reversal, reserving epididymectomy and orchiectomy as last-line treatments [8].

Few respondents utilized the validated COSI questionnaire, which offers a comprehensive assessment of symptoms, sexual function, and quality of life to better evaluate the impact of CSCP [1, 23]. However, its limited use may be related to time constraints, lack of familiarity, or clinical futility. Wider adoption of COSI may still be valuable to standardize outcome measurements, especially in a research setting. Given the complexity of managing patients with CSCP, the use of additional tools to assess clinical severity and guide treatment may aid both providers and patients. The use of the COSI questionnaire may be a powerful tool to set patient expectations and provide them with objective pre- and post-intervention metrics. Although not specifically named, the AUA endorse the use of validated questionnaires in the evaluation of CSCP [8].

Our study is not without limitations. As a survey-based study, our findings are susceptible to recall bias. Also, urologists with a greater interest in CSCP or increased exposure to it may have been more likely to complete the survey, leading to possible selection bias. As such, the responses may not represent the experiences or opinions of the broader US population of urologists. The survey did not assess the geographic distribution of respondents, which limited our ability to evaluate regional variation in practice patterns. Additionally, we did not differentiate between academic and community practice settings, which may further impact generalizability. While outcomes data were collected, they reflected physician perceptions of patient improvement rather than direct patient-reported outcomes, and questions regarding treatment success did not delineate between specific surgical or conservative modalities. Although we asked whether respondents used cord blocks for diagnostic and/or therapeutic purposes, we did not further differentiate the specific preparations used for each indication. This may have introduced variability in how respondents interpreted and answered the question, potentially skewing our results. Furthermore, we did not assess whether self-pay for vasectomy reversals influences a provider’s likelihood of offering this treatment. This financial consideration may significantly affect clinical decision-making and should be addressed in future studies. Finally, as a relatively brief survey, not all relevant aspects of CSCP diagnosis and management could be addressed.

Despite these limitations, this survey still effectively captures a comprehensive and meaningful cross-sectional view of current practice patterns in the treatment of CSCP. By including fellowship-trained experts, we ensured a high level of specialized knowledge that can inform future research. These data also provide valuable insight into real-world practice patterns as urologists begin to integrate the newly released AUA guidelines into their practice.

## Conclusion

Our study provides a contemporary assessment of practice patterns among reproductive urologists in the treatment of CSCP. Wide variability was found to exist in both diagnostic and treatment approaches. However, surgery was consistently associated with a higher rate of successful outcomes when compared to conservative management. These findings highlight areas of alignment and divergence with the 2025 AUA CSCP guidelines, suggesting a need for further comparative trials and real-world implementation studies.

## Supplementary information

List of questions provided to respondents

## Data Availability

The data generated during this study can be found within the published article or can be made available from the corresponding author on reasonable request.
