# Supplementary material for: Contemporary approaches and treatment perspectives for chronic scrotal content pain: insights from a national practice patterns survey of reproductive urologists
Source: Int J Impot Res. 2025 Jun 4;38(2):86–92. doi: 10.1038/s41443-025-01101-x (PMC12893908; doi:10.1038/s41443-025-01101-x)
Supplement: Supplementary file 1 — List of questions provided to respondents [file 41443_2025_1101_MOESM1_ESM.pdf]

Supplementary Figure 1. List of questions provided to respondents

---

1. What is your current level of training?

a) Medical Student
b) Urology Resident (please specify PGY level)
c) Fellow (please specify subspecialty, e.g., Andrology/Male Reproductive Medicine and Surgery,
d) Urologic Oncology, etc.)
e) Practicing General Urologist
f) Practicing Urologist with Subspecialty Focus (e.g., Andrology/Reproductive Medicine/Infertility)
g) Other (please specify)

---

2. How many years have you been practicing as a urologist?

a) Less than 1 year
b) 1-5 years
c) 6-10 years
d) 11-20 years
e) More than 20 years

---

3. Are you board-certified in urology?

a) Yes
b) No
c) Currently in the process of certification

---

4. Approximately how many patients do you see per year for chronic orchialgia?

a) Less than 10
b) 10-49
c) 50-100
d) Over 100

---

5. What percentage of your chronic orchialgia referrals come from the following sources? (Percent totals must equal to 100%)

a) Primary care
b) Emergency department or urgent care
c) Other urologist
d) Other physician (i.e. cardiologist, nephrologist, etc.)
e) Advanced practice provider (NP or PA)
f) Patient self-referral

---

6. For what percentage of chronic orchialgia patients are the following diagnostic studies ordered or physical exam maneuvers conducted by the referring provider prior to your evaluation? (Please provide a percentage for each option)

a) Urinalysis and/or urine culture
b) Scrotal ultrasound
c) Inguinal ultrasound
d) X-rays (hip, low back, etc.)
e) Advanced imaging (CT, MRI)
f) Semen analysis
g) Semen culture
h) Digital rectal exam
i) Hernia exam
j) Testicular tumor markers (ADH, LDH, HCG)
k) STI Testing
l) Other (please specify)
m) None of these

---

7. Which initial diagnostic studies or physical exam maneuvers do you perform more than 50% of the time for your index patients presenting with chronic orchialgia? (Select all that apply)

a) Urinalysis and/or urine culture
b) Scrotal ultrasound
c) Inguinal ultrasound
d) X-rays (hip, low back, etc.)
e) Advanced imaging (MRI, CT)
f) Semen analysis
g) Semen culture
h) Digital rectal exam
i) Hernia exam
j) Testicular tumor markers (AFP, LDH, HCG)
k) Advanced infection testing (e.g. STI testing, Mycoplasma/Ureaplasma, etc.)
l) Scrotal exam
m) No testing
n) Other (please specify)

---

8. Approximately how many of your patients are found to have idiopathic chronic orchialgia (meaning no identifiable cause can be found)? (Please use a sliding scale to indicate the percentage).

---

9. Which of the following conservative treatments do you commonly recommend after initial consultation and evaluation? (Rank the following treatments from most often

implemented to least often implemented after your initial consultation and evaluation for chronic orchialgia. You can click and drag each option.)

a) NSAIDs
b) Opioid pain medications
c) Antibiotics
d) Tricyclic antidepressants
e) GABA analogue
f) Physical therapy
g) Underwear with scrotal support
h) Reassurance
i) Other (please specify)
j) None

---

10. Please estimate the following for patients who undergo only conservative management (non-surgical treatment): (Please indicate a percentage for each option)

a) Percent of patients who report complete resolution of their pain
b) Percent of patients who report partial resolution of their pain
c) Percent of patient who report no resolution of their pain
d) Percent of patients who do not return for follow-up

---

11. How often do you refer patients to other specialists for chronic orchialgia? (Please indicate a percentage for each option)

a) Physical therapist (Pelvic floor physical therapy)
b) Psychiatrist
c) Chronic pain specialist
d) Another urologist
e) General surgeon
f) Other (please specify)

---

12. How often do patients who first had conservative treatment ultimately proceed with a surgical intervention? (Please use a sliding scale to indicate the percentage)

---

13. If you perform surgical interventions for chronic orchialgia in your practice, what surgeries do you typically perform? (Check all that apply)

a) Microsurgical denervation of the spermatic cord
b) Epididymectomy
c) Varicolectomy
d) Vasectomy reversal
e) Orchiectomy
f) Other (please specify)
g) I do not perform surgical interventions for chronic orchialgia

14. For post-vasectomy pain which surgical procedure are you most likely to perform?

a) Microsurgical denervation of the spermatic cord
b) Epididymectomy
c) Vasectomy reversal
d) Other (please specify)
e) I do not perform surgical procedures for post-vasectomy pain

---

15. Regarding vasectomy reversals for chronic orchialgia, what percent of patients are treated on a cash-pay basis (versus covered by insurance)? (Please use a sliding scale to indicate the percentage)

---

16. Regarding microsurgical denervation, what percent of your cases are: (Please use a sliding scale to indicate the percentage)

a) Unilateral
b) Bilateral

---

17. Please estimate the following for patients who undergo surgery:

a) Percent of patients who report complete resolution of their pain
b) Percent of patients who report partial resolution of their pain
c) Percent of patients who report no resolution of their pain
d) Percent of patients who do not return for follow-up

---

18. How do you measure the success of treatment in patients with chronic orchialgia? (Select all that apply)

a) Pain scores
b) Patient-reported outcomes
c) Validated Surveys (e.g., Chronic Orchialgia Symptom Index or COSI)
d) Decreased need for pain medication
e) Other (please specify)

---

19. In what percent of patients do you perform spermatic cord nerve block for chronic orchialgia? (Please use a sliding scale to indicate the percentage)

---

20. In what percent of patients do you perform spermatic cord nerve block as a treatment modality (versus solely for diagnostic evaluation)? (Please use a sliding scale to indicate the percentage)

---

21. Do you include a steroid in your nerve block cocktail (e.g., triamcinolone)?

a) Yes
b) No

---

22. Do you include a long-acting anesthetic in your nerve block cocktail?

a) Yes
b) No

23. Do you include a short-acting anesthetic in your nerve block cocktail?

a) Yes
b) No

---

24. What challenges do you face in managing patients with chronic orchialgia? *Optional

---

25. What areas of chronic orchialgia management do you believe require more research? *Optional
